# Overexpression of DNMT3b target genes during Enteric Nervous System development contribute to the onset of Hirschsprung disease

**DOI:** 10.1038/s41598-017-06539-8

**Published:** 2017-07-24

**Authors:** Leticia Villalba-Benito, Ana Torroglosa, Raquel María Fernández, Macarena Ruíz-Ferrer, María José Moya-Jiménez, Guillermo Antiñolo, Salud Borrego

**Affiliations:** 1 Department of Genetics, Reproduction and Fetal Medicine, Institute of Biomedicine of Seville (IBIS), University Hospital Virgen del Rocío/CSIC/University of Seville, Seville, 41013 Spain; 2 Centre for Biomedical Network Research on Rare Diseases (CIBERER), Seville, 41013 Spain; 3 Department of Pediatric Surgery, University Hospital Virgen del Rocío, Seville, 41013 Spain

## Abstract

Hirschsprung disease (HSCR) is attributed to a failure of neural crest cells (NCCs) to migrate, proliferate, differentiate and/or survive in the bowel wall during embryonic Enteric Nervous System (ENS) development. ENS formation is the result from a specific gene expression pattern regulated by epigenetic events, such DNA methylation by the DNA methyltransferases (DNMTs), among other mechanisms. Specifically, DNMT3b *de novo* methyltransferase is associated with NCCs development and has been shown to be implicated in ENS formation and in HSCR. Aiming to elucidate the specific mechanism underlying the DNMT3b role in such processes, we have performed a chromatin immunoprecipitation coupled with massively parallel sequencing analysis to identify the DNMT3B target genes in enteric precursor cells (EPCs) from mice. Moreover, the expression patterns of those target genes have been analyzed in human EPCs from HSCR patients in comparison with controls. Additionally, we have carried out a search of rare variants in those genes in a HSCR series. Through this approach we found 9 genes showing a significantly different expression level in both groups. Therefore, those genes may have a role in the proper human ENS formation and a failure in their expression pattern might contribute to this pathology.

## Introduction

Hirschsprung disease (HSCR, OMIM 142623) is a human developmental disorder, affecting 1:5,000 newborns. HSCR is characterized by the lack of enteric ganglia along the distal bowel, which causes a severe intestinal dysfunction^1^. The disease appears either sporadically or on a familial basis and may be associated with other developmental defects. Such aganglionosis observed in HSCR is due to failures in the proliferation, migration, differentiation and/or survival of the enteric precursors derived from neural crest cells (NCCs) which avoid a proper colonization of the gastrointestinal tract during embryonic development of Enteric Nervous System (ENS). HSCR phenotype is classified based on the length of the aganglionic region as: short-segment (S-HSCR) which includes patients with aganglionosis as far as the splenic flexure, long-segment (L-HSCR) in which aganglionosis extends beyond the splenic flexure and total colonic aganglionosis forms (TCA)^2^.

ENS development is a complex process that is regulated by a specific gene expression pattern at each stage during embryogenesis and any change in this process leads to serious consequences as observed in HSCR^3^. HSCR is a disorder with complex genetic basis, whose etiology is frequently due to the presence of several genetic variants acting in an additive or multiplicative manner^2, 4^. In this sense, the *RET* proto-oncogene (OMIM 164761) is the main gene associated with HSCR although up to another 40 different genes have been also related to the disease^5, 6^. *RET* coding rare mutations account for up to 50% of familial cases and for 10–20% of sporadic cases, while 5% of cases are due to mutations in genes other than *RET*. Therefore, it is necessary to continue with the search of additional genes to better delineate the HSCR etiology.

One of the well established mechanisms in the regulation of gene expression is epigenetics. Epigenetic mechanisms are acquiring increasing evidence of playing a major role in the onset of HSCR^7, 8^. Different epigenetic mechanisms are known, such as DNA methylation by the DNA methyltransferases (DNMT1, DNMT3a and DNMT3b). DNMT1 represents the maintenance methyltransferase and DNMT3a and 3b act as *de novo* methyltransferases establishing the methylation pattern during embryogenesis. It has been shown that both *de novo* methyltranferases are essential for the suitable NCCs development^9–11^. DNMT3b has been described as the methyltransferase that regulates the initial steps of progenitor cell differentiation during embryogenesis^12^. In addition, it has been shown that the expression of *DNMT3b* is lower in EPCs from HSCR patients compared with controls and this aberrant expression leads to a decrease of the global DNA methylation^7^, which may be contributing to an inappropriate gene expression pattern and to the onset of this pathology.

Therefore, we have performed a Chromatin immunoprecipitation coupled with massively parallel sequencing analysis (ChIP-seq) on mouse EPCs for the identification of DNMT3b target genes and then we have compared their expression levels in postnatal human EPCs from HSCR patients and controls. In addition, we have searched for rare variants into those target genes on whole exome sequences from a set of HSCR patients.

## Results

### Identification of DNMT3b-targets in mouse EPCs

**Figure 1:**
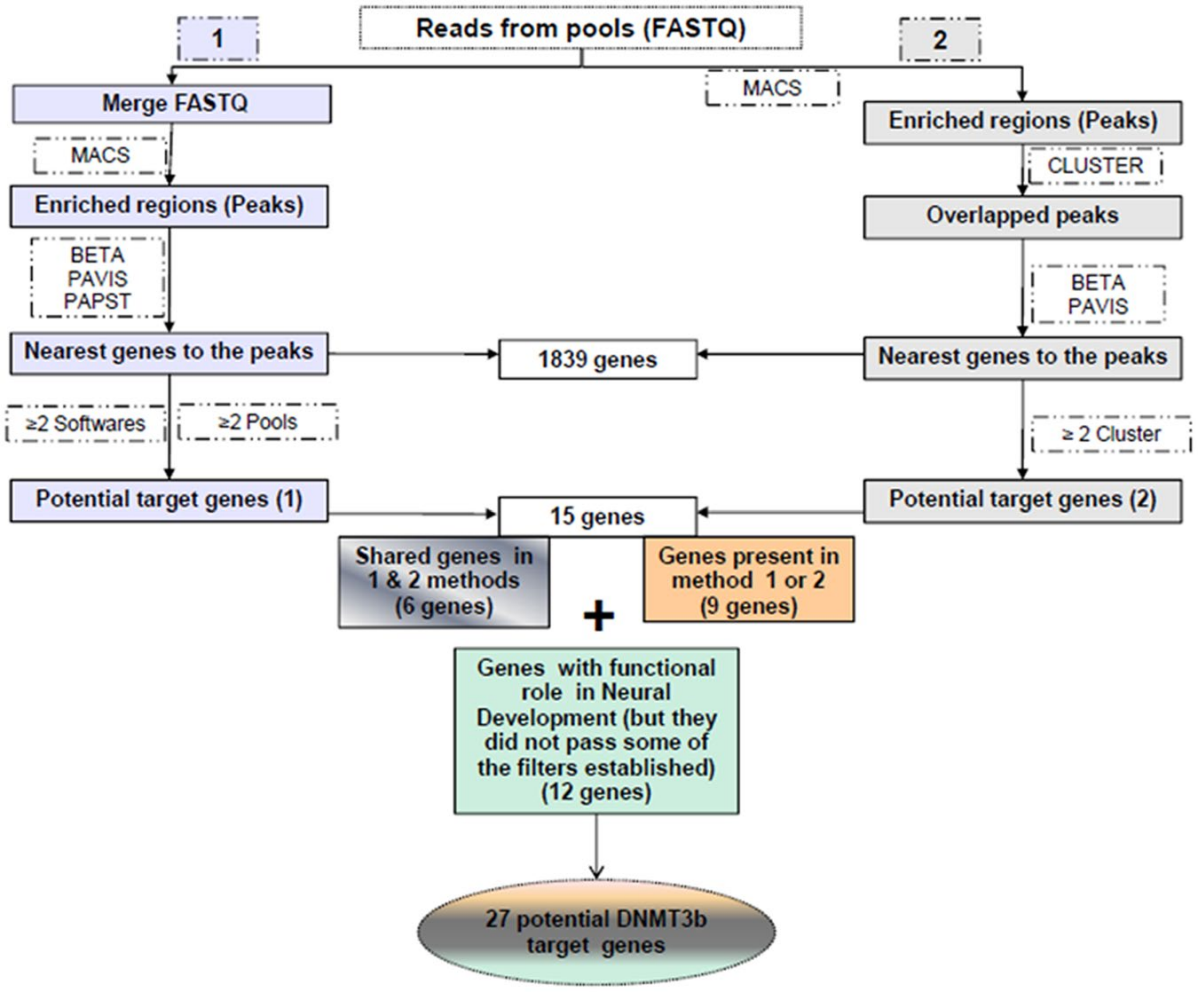
Prioritization and selection of DNMT3b-targets. Diagram showing both methodological approaches used for the selection of DNMT3b target genes.

**Figure 2:**
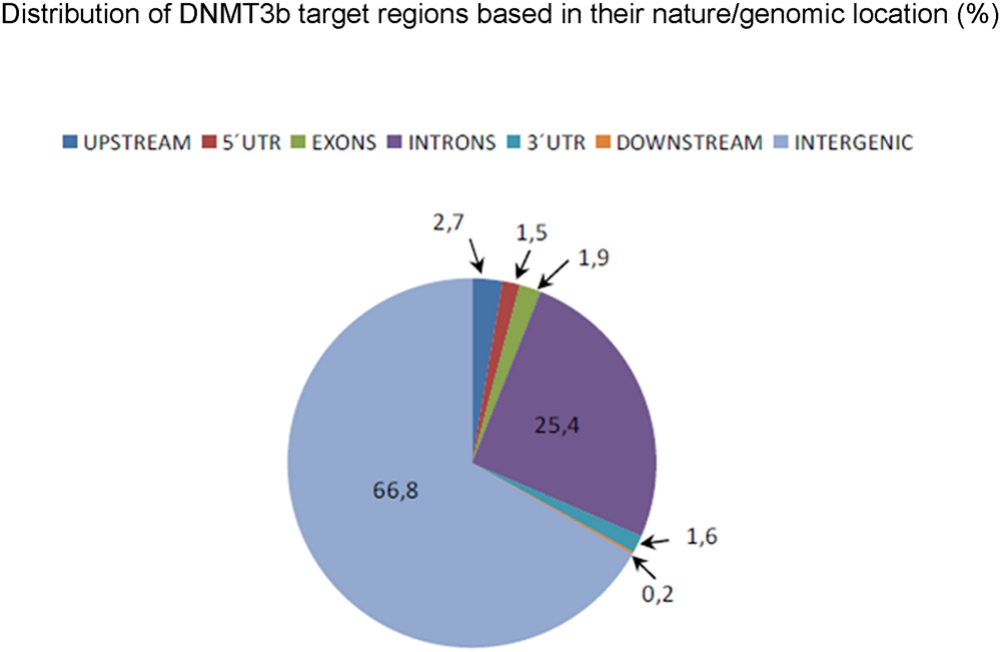
Peak distribution obtained in DNMT3b ChIP-seq assay. Diagram showing the peaks distribution, in which it is revealed that DNMT3b binding sites are preferentially intergenic and intronic regions.

ChIP-seq assay was performed in 3 different pools in order to identify a set of DNMT3b target genes in mouse EPCs. A total of 23,5 × 10^6^ (94%) reads passed the filters established by the MiSeq System and 12 × 10^6^ (51%) mapped reads were obtained. Two different analyses were subsequently used in order to select a number of reliable genes as targets of DNMT3b. Both methodological approaches are shown in Fig. 1 and described in detail in the methods section. Through the methods 1 and 2 we identified 924 and 152 genomic regions significantly enriched (peaks) respectively. In general, the analysis of the peaks distribution revealed that DNMT3b binding sites are preferentially intergenic and intronic regions (Fig. 2). Overall, taking into account the results from the 2 methods, a total of 15 different genes were obtained as potential Dnmt3b-targets (6 of them detected with both approaches). In addition we also identified another 12 genes corresponding to some of the observed peaks that had not passed any of the established filters but had been previously related with either the NCCs and/or the ENS development or with HSCR (Table 1).

**Table 1:** Selected genes in mouse-NLBS.

ANALYSIS METHODS	GENES	
Shared genes in 1 & 2 method (6 genes)	***Rn45s***	**Dnmt3b target genes**
	***Dpp9***	
	***Mydgf***	
	***Myo7a***	
	***Ano2***	
	***Gm17644***	
Genes present in 1 or 2 method (9 genes)	***Sulf1***	
	***2210408I21Rik***	
	***Ly75***	
	***Kpna1***	
	***4933422A05Rik***	
	***Chl1***	
	***Cyr61***	
	***Gphn***	
	***Bbx***	
Genes with functional role in Neural Development (but they did not pass some of the filters established) (12 genes)	***Rab10os***	
	***Drg1***	
	***Eif4enif1***	
	***Sfi1***	
	***Ppp2r2b***	
	***Dopey2***	
	***Smo***	
	***Litaf***	
	***Cdk5rap2***	
	***Limd1***	
	***4921504E06Rik***	
	***Tmem125***	

### Genes selected for the assessment of their potential implication in HSCR

To further investigate if the 27 genes selected as potential Dnmt3b target genes in mouse have any kind of association with the onset of HSCR, we searched the Human Orthologs of these genes in the Mouse Genome Informatics database (MGI). Specifically, the *Rn45s* gene was not found in MGI but we could find the *RN45S* gene, also known as *RNA45S5*, in the GeneCards Human Gene Database. The Human Orthologs of some target genes are still unknown and for that reason we searched their homologous sequence using the Basic Local Alignment Search Tool (BLAST) and selected those human genes with the highest similarity (Table 2). The human pseudogene *RP11 220D10.1* appeared as the region with the highest similarity (82.44%) with the mouse gene *4933422A05Rik*. The pseudogenes are the result of reverse mRNAs transcripts from original genes that are integrated into a new region in the genome. Therefore, the analysis of these genomic regions are complicated and we decided to eliminate *RP11 220D10.1* from de analysis. At this point, we counted 26 genes in humans.

**Table 2:** Homologous genes in Human.

Genes (mice)	Ortholog genes in Human
***Rn45s***	***RNA45S5***
***Gm17644***	***LINC01603*** * (79,27%)
***2210408I21Rik***	***KIAA0825***
***Rab10os***	***RAB10*** * (96,28%)
***4921504E06Rik***	***C10ORF67***

*Orthologs unknown, genes selected by sequence similarity (%) through BLAST tool.

In terms of DNMT3b target genes we also found it interesting to investigate about their signaling pathways and interaction with other genes as well as about their function. With this aim, we used the Ingenuity Pathway Analysis (IPA) powerful tool, which enabled us to associate physiological functions and diseases to our target genes and obtain a high level overview of their biological interactions described so far. Based on all the genetic interactions obtained for each target gene with the parameters established in the IPA (see Supplementary Fig. S1), we decided to select an additional group of genes (*EED, STAT3, NEUROG1, ADAM8, NEDD4* and *NEDD4L*), because either their interaction with more than one DNMT3b target gene or their function in “cellular development”. Therefore, we finally considered a total of 32 genes to perform an analysis of their expression patterns in human EPCs (Table 3).

**Table 3:** Genes included in the differential expression study in human NLBs.

ASSAY	GENE	NAME	SELECTION
***TaqMan® Array Plate***	*DPP9*	Dipeptidyl Peptidase 9	**DNMT3b Target genes**
	*MYO7A*	Myosin VIIA	
	*CDK5RAP2*	CDK5 Regulatory Subunit Associated Protein 2	
	*MYDGF*	Myeloid-Derived Growth Factor	
	*SULF1*	Sulfatase 1	
	*ANO2*	Anoctamin 2	
	*CHL1*	Cell Adhesion Molecule L1 Like	
	*LIMD1*	LIM Domains Containing 1	
	*CYR61*	Cysteine Rich Angiogenic Inducer 61	
	*GPHN*	Gephyrin	
	*SFI1*	SFI1 Centrin Binding Protein	
	*DOPEY2*	Dopey Family Member 2	
	*DRG1*	Developmentally Regulated GTP Binding Protein 1	
	*SMO*	Smoothened Frizzled Class Receptor	
	*C10ORF67*	Chromosome 10 Open Reading Frame 67	
	*TMEM125*	Transmembrane Protein 125	
	*KIAA0825*	KIAA0825	
	*LY75*	Lymphocyte Antigen 75	
	*BBX*	Bobby Sox Homolog	
	*EIF4ENIF1*	Eukaryotic Translation Initiation Factor 4E Nuclear Import Factor 1	
	*PPP2R2B*	Protein Phosphatase 2 Regulatory Subunit B. Beta	
	*KPNA1*	Kayopherin Subunit Alpha 1	
	*LITAF*	Lipopolysaccharide Induced TNF Factor	
	*RAB10*	RAB10. Member RAS Oncogene Family	
	*EED*	Embryonic Ectoderm Development	**Related genes of DNMT3b target genes established by IPA**
	*NEDD4*	Neural Precursor Cell Expressed Developmentally Down-Regulated 4. E3 Ubiquitin Protein Ligase	
	*NEDD4L*	Neural Precursor Ceel Expressed, Developmentally Down-Regulated 4-Like. E3 Ubiquitin Protein Ligase	
	*STAT3*	Signal Transducer and Activator of Transcription 3	
	*NEUROG1*	Neurogenin 1	
	*ADAM8*	ADAM Metallopeptidase Domain 8	
	*S18*	Endogenous control	
***SYBR Green***	*LINC01603*	Long Intergenic NON-Protein Coding RNA 1603	**DNMT3b Target genes**
	*RNA45S5*	RNA, 45 S Pre-Ribosomal 5	
	*GAPDH*	Endogenous control	

### Analysis of differential expression patterns in HSCR vs Controls EPCs

**Figure 3:**
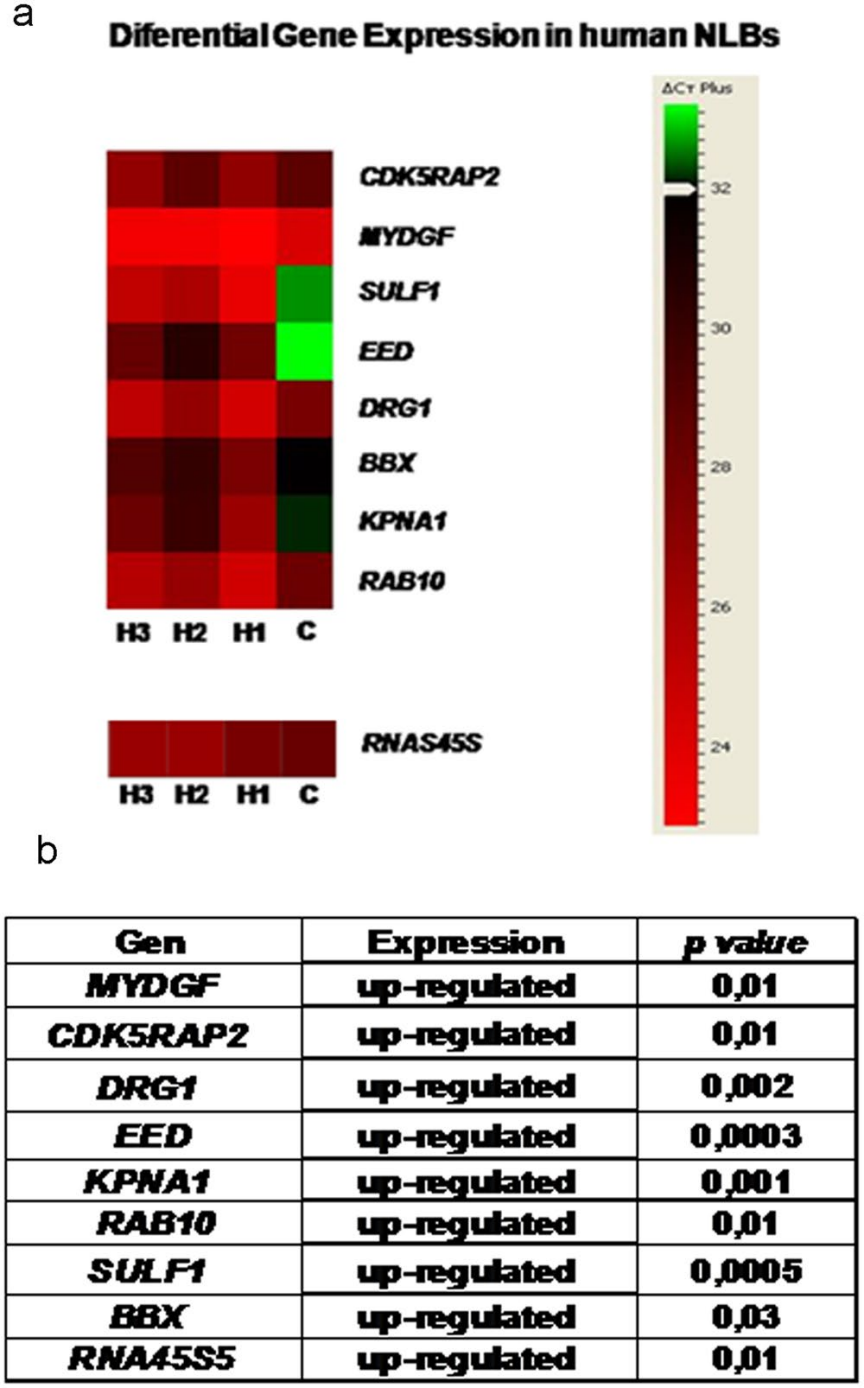
Differential gene expression in HSCR-NLBs *versus* Control-NLBs. (**a**) The Heat map was generated using DataAssist v3.0 software (Life Technologies) and it represents the messenger RNA expression levels of 9 genes expressed in colon tissue from HSCR patients and controls. Genes were hierarchically clustered by Pearson correlation coefficient using average linkage. The color scale, representing ΔCt, is shown on the right side. Green indicates genes with relatively decreased expression in HSCR, whereas red indicates genes with relatively increased expression in HSCR compared with the controls. Ct, cycle threshold; Hirschsprung patiens (H); Control (C). (**b**) Table showing the variations in the gene expression levels in the NLBs from HSCR patients compared with Controls.

To evaluate the possible involvement in HSCR of the 32 genes previously selected, we performed a differential gene expression study in human EPCs cultured as Neurosphere-like Bodies (NLBs) from HSCR patients compared with controls. As a result 12 genes showed a positive expression in human EPCs (see Supplementary Table S1). When we compared their gene expression pattern in EPCs from HSCR patients *versus* controls, 9 of the genes (*KPNA1, EED, SULF1, CDK5RAP2, BBX, DRG1, RAB10*, *MYDGF* and *RNA45S5*) were found to be significantly up-regulated in the HSCR group (p value ≤ 0,05) (Fig. 3). None of such target genes overlapped with those reported from prior Genome Wide Association/Expression Studies in the context of HSCR.

### Identification of rare variants possibly associated to HSCR in DNMT3b-targets and other related genes expressed in human EPCs

To assess whether the 14 selected genes shown to be expressed in human EPCs could contribute to HSCR susceptibility through rare sequence variants, we analysed the whole exome sequencing (WES) data from 56 HSCR patients^13^. A total of 12 heterozygous rare variants (10 SNVs and 2 small deletions) distributed among 5 of the 15 candidates were identified in 10 patients. Nine of the variants were located within the coding region and 3 were in untranslated regions (UTR). All of them were confirmed by Sanger sequencing and details are shown in Table 4.

**Table 4:** Rare variants identificated in exome sequencing from HSCR patients.

Gene	RefSeq	Location	Variants	rs	Patient ID^a^	Inheritance	Phenotype	1000G_MAF (phase 3) ALL	1000G_MAF (phase 3) EUR	EVS_MAF	ExAC_MAF	MGP_MAF	In silico prediction (SIFT /Polyphen2)
*BBX*	NM_020235	3:g.107466840 C > T; exon	c.779C > T:p.Ala260Val	rs150121801	3733^b^	Father	S-HSCR	0.0012	0.002	0.0012	0.0026	NA	T/B
		3:g.107435532 C > T; exon	c.241C > T:p.Arg81Trp	rs142400819	4086	Mother	S-HSCR (Familial)	0.0030	0.007	0.0030	0.0028	0,006	D/D
*SULF1*	NM_015170	8:g.70541785_70541787delAAC; exon	c.2155_2157delAAC:p.Asn720del	rs150178205	10943	Mother	L-HSCR	0.0038	N	0.0045	0.0009	0,002	Neutral/-
*RAB10*	NM_016131	2:g.26257138 C > T; UTR5	c.-340C > T	rs112783454	4949	Father	S-HSCR	0.0052	0.012^d^	N	N	0,008	
		2:g.26257109_26257110delGA;UTR5	c.-369_-368delGA	rs745876590	4462^b^	Father	S-HSCR	N^c^	N	N	N	NA	
*PPP2R2B*	NM_181674	5:g.145979904 C > T; exon	c.1108G > A:p.Val370Ile	rs369931023	3485	Father	S-HSCR	0.0002	N	0.00008	0.00006	NA	T/B
		5:g.146017897 T > C; exon	c.905A > G:p.Asn302Ser	rs150981315	3708	Father	S-HSCR	N	N	0.0003	0.0004	NA	D/B
		5:g.145969279 G > C; UTR3	c.*231C > G	rs141447016	3364	Mother	L-HSCR	0.0038	0.007	N	N	NA	
*CDK5RAP2*	NM_018249	9:g.123334309 G > T; exon	c.70C > A:p.Pro24Thr		3606	Father	S-HSCR	N	N	N	N	NA	T/B
		9:g.123216045 T > C; exon	c.2482 A > G:p.Lys828Glu	rs549081765	8079	Mother	S-HSCR	0.0004	N	N	0.0002	NA	T/B
		9:g.123253661 T > C; exon	c.1406 A > G:p.Asn469Ser	rs754779136	8079	Father	S-HSCR	N	N	N	0.000008	NA	T/B
		9:g.123287277 G > A; exon	c.1079 C > T:p.Thr360Ile	rs145165171	10943	Father	L-HSCR	0.001	0.004	0.001	0.0009	0,002	T/B

^a^patients with two different variants are in bold. ^b^Variants also present in one sibling. ^c^N means variant not present in the database (1000 Genomes, EVS, ExAC). ^d^MAF > 0.01 in European population. NA: Not Available.

The analysis revealed just one familial HSCR case carrying a rare variant in one of the genes, but showing no segregation with the phenotype since it was not present in the other affected member of the family and it had been inherited from his unaffected mother. The remaining variants were identified in sporadic HSCR cases, most of them with a short-segment phenotype. All these rare variants were inherited from one of their parents and occasionally they appeared in unaffected siblings. Interestingly, we observed that two of these sporadic patients carried two different rare variants inherited from each parent. One of them was a female L-HSCR patient carrying both variants in two different genes, *SULF1* and *CDK5RAP2*, while the other one was a male S-HSCR patient with two different rare variants in *CDK5RAP2*.

## Discussion

There are growing evidences indicating that epigenetic mechanisms have an essential role for the proper ENS and NCCs development^8^. In this sense, the *de novo* methyltransferase *DNMT3b* has been associated with HSCR^7^ and, therefore, we have focused in the identification of DNMT3b target genes to elucidate the pathways and genes which are regulated by this methyltranferase. Taking into account that in the context of rare disorders such as HSCR the recruitment of a high number of patients is really difficult, we designed an indirect experiment in EPCs from mouse to identify the possible Dnmt3b target genes by ChIP-seq, since with this kind of samples we have no limitations regarding the minimal starting quantity required for the assays. As a result, we observed that the majority of Dnmt3b binding sites were located in intronic and intergenic regions, from which *a priori* it is not easy to predict functional consequences. However, there are already some studies that have identified differentially methylated regions (DMRs), not exclusively in intragenic but also in intergenic regions, and proposed these non-promoter CpG islands as regulatory elements^14–16^. The roles of this DNA methylation remain unclear as well as how these regions are established, maintained, and function. Nevertheless, increasingly more studies are identifying the methylation of these regions as regulators of gene expression^17^.

Based in the results obtained in mouse, to continue with the investigation of the role of the resulting target genes in HSCR, we performed a differential expression study in human EPCs from HSCR patients and controls. It is worthy to note that all the genes observed to be differentially expressed in HSCR patients versus controls are up-regulated in the first group, which fits with the lower global DNA methylation due to the decreased expression of *DNMT3b* described in HSCR patients^7^. In addition, we carried out a search of rare variants in those genes in a HSCR series. Interestingly, the finding of rare variants with incomplete penetrance in 5 of these genes in our series of patients, suggest that they may also be contributing to the HSCR phenotype. Moreover, most of those genes have been related either directly or indirectly to NCCs development, ENS and Central Nervous System (CNS) formation, neurogenesis and/or HSCR. In the case of *MYDGF*, its involvement in those processes had not been described to date. This gene encodes for the Myeloid Derived Growth Factor, which promotes cardiac myocyte survival and angiogenesis^18^. Such cell processes are also essential for the correct ENS formation at the neuronal level.

*KPNA1* encodes for karyopherin alpha1, a protein that belongs to the importin-alpha family that is implicated in the STAT3 transport into the cell nuclei^19^. We have observed that *STAT3* is expressed in human EPCs and this signal transducer and transcription activator has been related with the maintenance of the NCCs in an undifferentiated state during development, promoting their differentiation when it is down-regulated^20^. Moreover, STAT3 pathway activation is triggered by the phosphorylation of the RET receptor^21^, which is encoded by the main gene involved in HSCR described so far^2, 5^. On the other hand, EED (Embryonic Ectoderm Development) is an epigenetic regulator that belongs to Polycomb repressive proteins and controls neural crest gene expression during NCCs specification and migration^22^. We decided to include *EED* in this study because Foust *et al*. described the interaction between EED and KPNA1^23^, also predicted by the IPA tool. Interestingly, we have observed that this gene is expressed in human EPCs and it is also found to be significantly up-regulated in EPCs from HSCR patients *versus* controls.

With respect to *SULF1*, it encodes for an endosulphatase that regulates the sulphation pattern of Heparan Sulphate Proteoglycans (HSPG). It has been described that *SULF1* is required for RET/GDNF interaction^24^. Activation of the RET pathway through GDNF promotes enteric NCCs proliferation and differentiation, essential for ENS formation and this pathway has been largely related with the onset of HSCR. Regarding *BBX*, it encodes for a transcription factor member of the SOX family. Several transcription factors belonging to this family have been associated with neural stem cells maintenance (*SOX9*)^25^, NCCs development (*SOX2* and *SOX8*) or HSCR (*SOX10 and SOX2*)^26–28^. Specifically, *BBX* is expressed in progenitor cells, regulates their self-renewal capacity and prevents their differentiation during CNS development^29^. To finish with this group of genes, we should highlight *DRG1* which maps to 22q12^q13.1 region^30^. It has been shown in humans that this locus is a susceptibility region for HSCR^31^. *DRG1* encodes for a GTPase highly expressed during CNS embryogenesis^32^.

On the other hand, we have identified *CDK5RAP2* and *RAB10*, both genes implicated in neurogenesis which is an important process for the ENS formation^33^. *CDK5RAP2* encodes for a CDK5 regulatory subunit associated protein 2 and is essential for the correct mitotic cell cycle. Mice with an in-frame deletion in *CDK5RAP2* show microcephaly and proliferative and survival defects in neurons during development. These mice also show smaller ganglionic eminences in comparison with the wild-type phenotype^34^. It has also been demonstrated that a loss of CDK5RAP2 function results in premature cell cycle exit, leading to a premature differentiation into neurons^33^. It is worth mentioning that two different *CDK5RAP2* rare variants (c.2482A > G:p.Lys828Glu and c.1406A > G:p.Asn469Ser) were identified in the same patient. In addition, we identified another HSCR patient who carries the variant p.Thr360Ile in *CDK5RAP2* and also the variant c.2155_2157delAAC:p.Asn720del in *SULF1*. This could suggest that these variants here identified may be contributing to the final phenotype, reinforcing the possible role of *CDK5RAP2* and *SULF1* in HSCR phenotype.

Regarding *RAB10*, it is a member of small GTPases *RAS* oncogene family. This gene is involved in early embryogenesis^35^ and its role in axon development and dendrite growth, essential for the proper neuronal networks formation in CNS, has been also shown^36, 37^.

Also of interest *RNA45S5* is a non coding RNA and it is a precursor for the 18S, 5.8S and 28S rRNA subunits. Sánchez-Martín *et al*. showed that *Rn45s* is expressed in postnatal mice CNS and it is regulated by methylation level^38^. On the other hand, *TCOF1* has been identified as a gene which plays a central role in the regulation of ribosome biogenesis and pre-ribosomal processing. Loss of function mutations in this gene cause Treacher Collins syndrome (TCS, OMIM 154500), that it is characterized by abnormal cranial neural crest cells, survival, migration and differentiation during development^39^. Therefore, there are evidences suggesting that the genes related with the ribosome biogenesis have a role in NCCs development.

In summary, we have performed a study on mouse EPCs which has allowed us to identify for the first time a set of DNMT3b target genes with a potential role in ENS development. Subsequently, we have analyzed the implication of these target genes in human EPCs, and this approach has led us to identify that their expression is regulated by DNMT3b-methylation in these cells during ENS formation. Therefore epigenetic mechanisms have acquired a relevant role in the HSCR context. Accordingly, we may conclude that these genes could be implicated in the proper ENS formation and failures at this regulation level may contribute to trigger the onset of this pathology. Therefore, the role of such genes must be further analyzed in detail in the context of HSCR. It is worthy to note that our findings support the convenience to focus the search of mechanisms involved in HSCR onset from an epigenetic point of view together with the conventional approaches.

## Material and Methods

### Culture of enteric neurosphere-like bodies (NLBs) from human and mouse

EPCs were obtained from human postnatal tissues of ganglionic gut in 14 sporadic non-related patients diagnosed with isolated HSCR (L-HSCR = 2; S-HSCR = 12, male: female = 11:3), as well as in 5 patients with other gastrointestinal disorders undergoing gut resection surgery at our Hospital that were used as controls (male:female = 3:2). For both HSCR patients and control individuals, age ranged from 3 months to 3 years. The written informed consent for surgery, clinical and molecular genetic studies was obtained from all the human participants or their guardians. The study was approved by the Ethics Committee for clinical research of the University Hospital Virgen del Rocío (Seville, Spain) and complies with the tenets of the declaration of Helsinki.

EPCs were extracted from ganglionic bowels of both CD-1 7days-aged mice and humans, and were grown as Neuroesphere-like bodies (NLBs) as described elsewhere^7^. All the procedures involving mice were performed in accordance with the European Union guidelines (2010/63/EU) and the Spanish Law (R.D. 53/2013 BOE 34/11370-420, 2013) concerning the care and use of laboratory animals, and were approved by the Animal Experimentation Ethics Committee (EAEC/IEC) of University Hospital Virgen del Rocío/Institute of Biomedicine of Seville (IBIS).

### Chromatin immunoprecipitation (ChIP)

ChIP assay was performed using chromatin from fixed EPCs derived from mouse-NLBS (10^6^) in 1% formaldehyde and was sonicated in a Bioruptor System (Diagenode, USA) which let us to obtain DNA fragments between 200 and 1000 bp in size. ChIP assay was performed with the Magna Chip HiSens Kit (Millipore,USA), according to manufacturer’s instructions and using the Anti-Dnmt3b antibody-ChIP Grade (Abcam, USA). 10% of samples volume/reaction was kept as controls (input) before immunoprecipitation. ChIP and input specimens were quantified by Quant-iT Picogreen dsDNA Assay Kit (ThermoFisher Scientific, USA).

### Sequencing

ChIP and input samples were processed for subsequent sequencing with the ChIP-Seq DNA sample prep kit (Illumina, USA), following the manufacturer’s instructions. Size-selection was then performed by agarose gel electrophoresis to isolate fragments with size between 200 and 300bp. Afterwards, purified libraries were amplified by PCR and size range was subsequently checked in the Agilent 2100 Bioanalyzer using the High Sensitivity DNA Kit (Agilent, USA). Quantification was performed in the 7500-Real Time PCR System (Applied Biosystems) with the Library Quantification Kit (Kapa Biosystems). After proper dilutions, 4 nM libraries were processed, to obtain pools at a final concentration of 8 pM following the instructions of the Miseq Reagent kit V2. (50 cycles) (Illumina, USA). Finally we proceeded to sequence a total of 3 pools accounting for 12 ChIP and 12 inputs in the MiSeq System (Illumina, USA).

### Sequencing data analysis

After filtering, mapping was performed using Bowtie tool (mouse genome: mm9). MACS (Model-based Analysis of ChIP-Seq) algorithm was used for calling peaks, and BETA (Binding and Expression Target Analysis) minus software was applied to assign the ChIP-seq peak data to the neighboring genes (annotation). Such tools are available in the Galaxy and/or Galaxy/Cistrome platforms (https://usegalaxy.org/; http://cistrome.org/ap/root). In combination with BETA minus, we also used PAVIS (http://manticore.niehs.nih.gov/pavis2/) and PAPST tools, (https://github.com/paulbible/papst) to select and prioritize the candidate DNMT3b target genes.

Two different analyses were used to obtain a feasible and reliable number of Dnmt3b target genes. In the method 1, we merged all the FASTQ files from the ChIPs within the same pool and using the MACS software. Then through the BETA minus, PAVIS and PAPST tools we identified the genes located nearest to the regions represented by those peaks. Finally we selected the genes obtained after the analyses of at least 2 of the 3 softwares in at least 2 of the 3 pools. In the method 2, the peaks were identified with the MACS software using individually the FASTQ files from each ChIP within the same pool. Next, we applied the cluster tool to obtain the overlapping peaks and BETA and PAVIS software were used to identify the nearest genes. At last, we selected the genes that were identified in at least 2 of the clusters. Both methodological approaches are shown in a diagram in the Fig. 1.

All raw and peak data obtained in the study can be found in Gene Expression Omnibus (GEO), the following link has been created to allow review of record GSE92942 while it remains in private status: https://www.ncbi.nlm.nih.gov/geo/query/acc.cgi?token=yvmriecgjbyldqx&acc=GSE92942.

### Identification of DNMT3b target genes in humans and their gene interaction

We have searched the Human Orthologs of the Dnmt3b target genes selected in mouse EPCs, by using the MGI database (http://www.informatics.jax.org) that provides integrated genetic, genomic and biological data for the study of human health and disease. When the orthologs are unknown we used the BLAST (https://blast.ncbi.nlm.nih.gov/Blast.cgi). Gene interactions of the DNMT3b target genes were obtained through the following parameters established by us in IPA tool: Genes and Chemicals; Interaction network (human, mouse, rat); Path designer; Overlay tool: Function & Disease: “Cellular development”. IPA led us to acquire of the interaction network of each DNMT3b target gene, where the genes involved in “cellular development” with a p-value cutoff of 0.05 were labeled.

### Gene Expression Study by quantitative reverse transcription- PCR (RT- qPCR)

We carried out a differential expression study of the resulting DNMT3b target genes previously identified in NLBs cultures, as well as of a set of genes related with them which were determined using the IPA tool. Finally we obtained 32 genes to assess their pattern expression in EPCs from both HSCR patients and controls. Purification and synthesis of cDNA were performed using the protocol provided by µMACS mRNA isolation Kit and µMACS cDNA Synthesis Kit in a thermo MAKSTM Separator (MACS Miltenyi Biotech, Germany) or RNAeasy Micro kit and RT^2^ First Strand kit (Qiagen, Germany). Expression studies were carried out in an Applied Biosystems 7900HT system (Life Technologies, USA) through the TaqMan® Array Plate (Life Technologies, USA) and SYBR Green methods (LincRNA and rRNA gene) (Bio-Rad, USA). Analysis was performed using the RQ Manager Software (Life Technologies, USA). *18S* or *GAPDH* were used as endogenous controls respectively (Supplementary Table 1). Following the software recommendations, the upper limit of the cycle threshold (Ct) was set to be 32 for the TaqMan Array Gene Signature Plates and 35 for the SYBR Green assay. We considered positive expression exclusively when Ct values were lower than such values.

### Sequence variants analysis

In order to identify potential disease-causing variants in the genes selected after expression analysis, we used the data from a WES study previously published by our group^13^ that included 56 HSCR patients comprising 39 sporadic and 17 familial cases. Single Nucleotide Variants (SNVs) and short insertions or deletions (Indels) were annotated using Annovar (hg19 Refgene) (http://wannovar.wglab.org/index.php) and only those variants with a minor allele frequency (MAF) value < 0.01 in at least one databases (1000 Genomes, Exome Variant Server, Exome Aggregation Consortium or dbSNP) were selected. Additionally, the MAF was also checked in the 267 Spanish healthy phenotype exomes sequenced in the context of the Medical Genome Project^40^. The pathogenicity of SNVs was predicted by PolyPhen-2 (http://genetics.bwh.harvard.edu/pph2/) and SIFT (http://sift.bii.a-star.edu.sg) algorithms. Selected variants were further confirmed by Sanger sequencing.

### Statistical analyses for gene expression study

Data are presented as the mean ± SEM (Standard Error Mean) of values obtained from at least three experiments. Comparisons between values obtained in Control-NLBs and HSCR-NLBs were analyzed using the Student’s t test. Differences were considered significant when p value ≤ 0.05.

## Electronic supplementary material

Supplementary Information
