# Supplementary material for: Overexpression of DNMT3b target genes during Enteric Nervous System development contribute to the onset of Hirschsprung disease
Source: Sci Rep. 2017 Jul 24;7:6221. doi: 10.1038/s41598-017-06539-8 (PMC5524929; doi:10.1038/s41598-017-06539-8)
Supplement: Supplementary file 1 — Supplementary Information [file 41598_2017_6539_MOESM1_ESM.pdf]

SUPPLEMENTARY INFORMATION

TITLE: Overexpression of DNMT3b target genes during Enteric Nervous System development contribute to the onset of Hirschsprung disease.

AUTHORS: Leticia Villalba-Benito[1,2,#], Ana Torroglosa[1,2,#], Raquel María Fernández[1,2], Macarena Ruíz-Ferrer[1,2], María José Moya-Jiménez[3], Guillermo Antiñolo[1,2], Salud Borrego [1,2,]*.

[#] These authors contributed equally.

AFFILIATIONS:

[1] Department of Genetics, Reproduction and Fetal Medicine. Institute of Biomedicine of Seville (IBIS), University Hospital Virgen del Rocío/CSIC/University of Seville, Seville, 41013, Spain.

[2] Centre for Biomedical Network Research on Rare Diseases (CIBERER), Seville, 41013, Spain.

[3] Department of Pediatric Surgery, University Hospital Virgen del Rocío, Seville, 41013, Spain.

***CORRESPONDING AUTHOR:**

Salud Borrego, MD, PhD.

Department of Genetics, Reproduction and Fetal Medicine.

University Hospital Virgen del Rocío.

Avda. Manuel Siurot s/n. 41013 Seville, Spain

Voice: + 34 955 012780, Fax: + 34 955 012779

e-mail: salud.borrego.sspa@juntadeandalucia.es

Table S1: Genes expressed in human enteric precursor cells.

Gene	Ct	Function
MYDGF	26	It is associated with several biological processes such as angiogenesis stimulating endothelial cell proliferation.
CDK5RAP2	30	It is essential for a correct mitotic cell cycle.
DRG1	29	It has a potential role in proliferation and/or cell differentiation during development of nervous system.
EED	32	It is major component of Polycomb repressive complex 2, this is a epigenetic regulator that methylates histone H3 at lysine 27, controling neural crest gene expression during neural crest development
KPNA1	31	It plays a key role in the nuclear transport of signal transducer and activator of transcription 3 (STAT3) and STAT1 transcription factors.
LITAF	27	Its main functions are inflammatory response and apoptosis.
RAB10	30	It is implicated in vesicle trafficking. These events mediated vesicle trafficking by Rab10, are essential for axon development which is essential for proper neurons morphogenesis and thus for correct neuronal migration, and for dendrite growth and morphogenesis which is necessary for connectivity between neurons and to capture information from the environment.
STAT3	30	It mediates the expression of a variety of genes in response to cell stimuli, and thus plays a key role in many cellular processes such as cell growth and apoptosis.
SULF1	32	It controls the neuronal and glial generation during ventral neural tube development. Also it is essential for normal migration of neural crest cells.
BBX	28	It has been associated with maintaining neural stem cells, neural crest cells development and progenitor cell selfrenewal.
PPP2R2B	31	It is widely expressed in embryonic mouse brain. At the cellular level it is limited to neurons and it plays a role in neuronal cell during brain development
RNA45s5	29	It is a non coding RNA which serves as the precursor for the 18S, 5.8S and 28S

Figure S1

SULF1

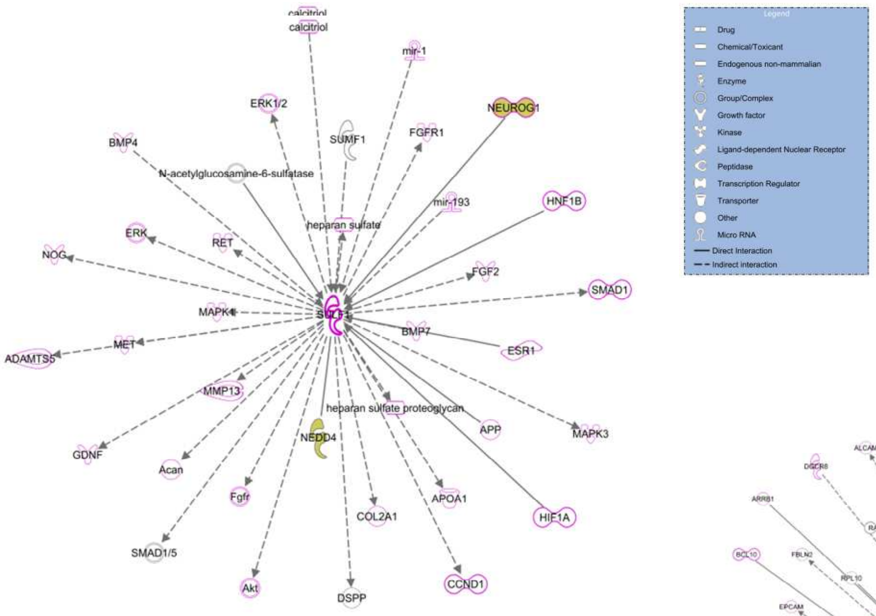

SMO

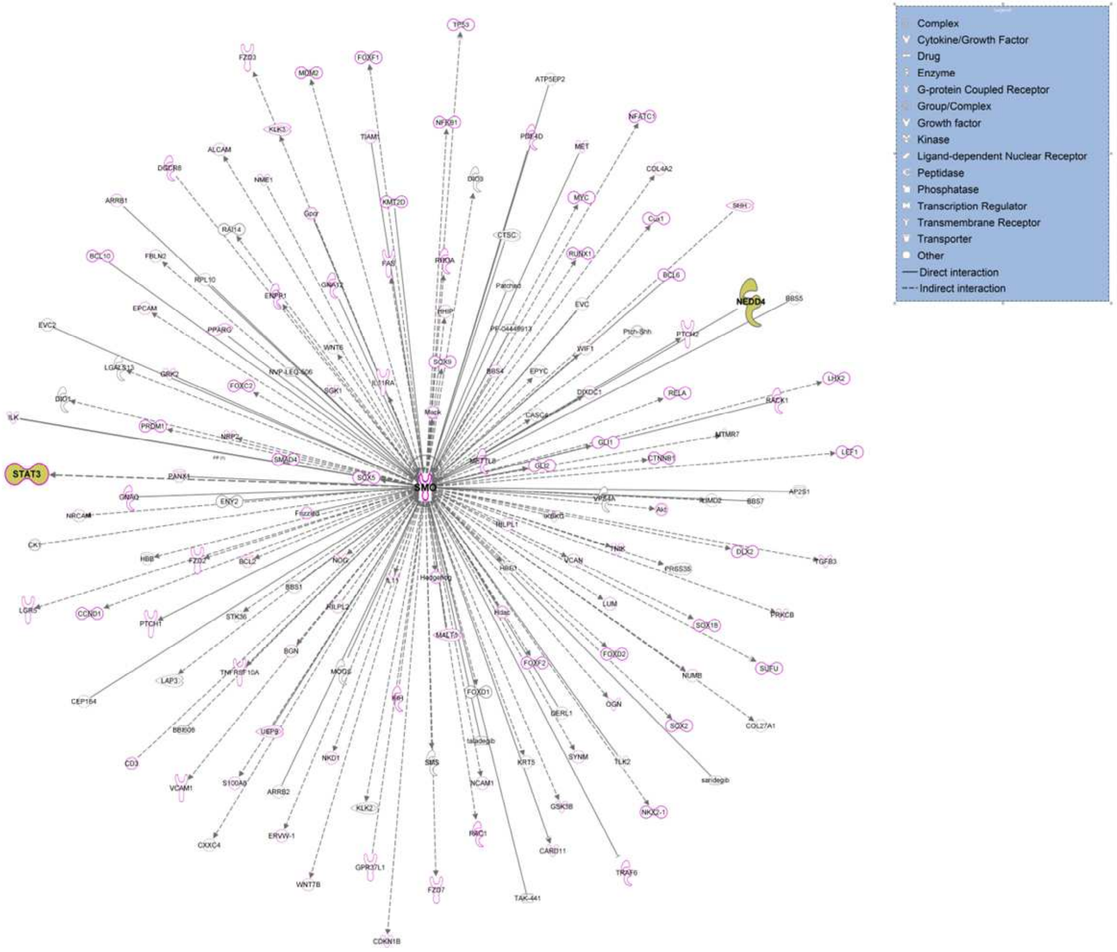

KPNA1

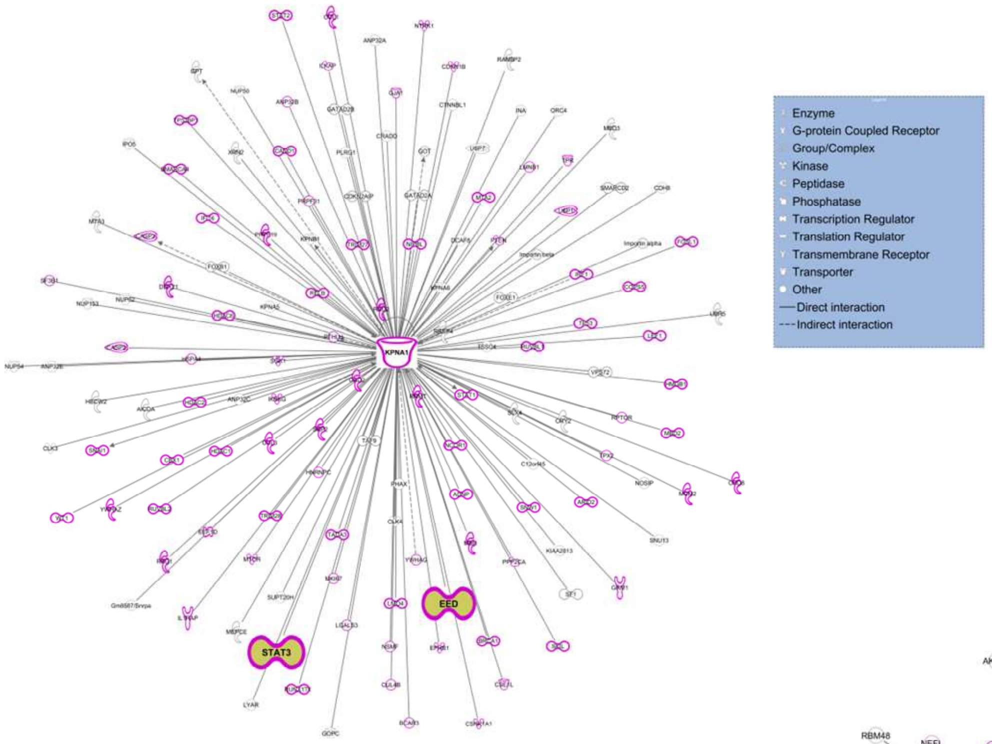

CDK5RAP2

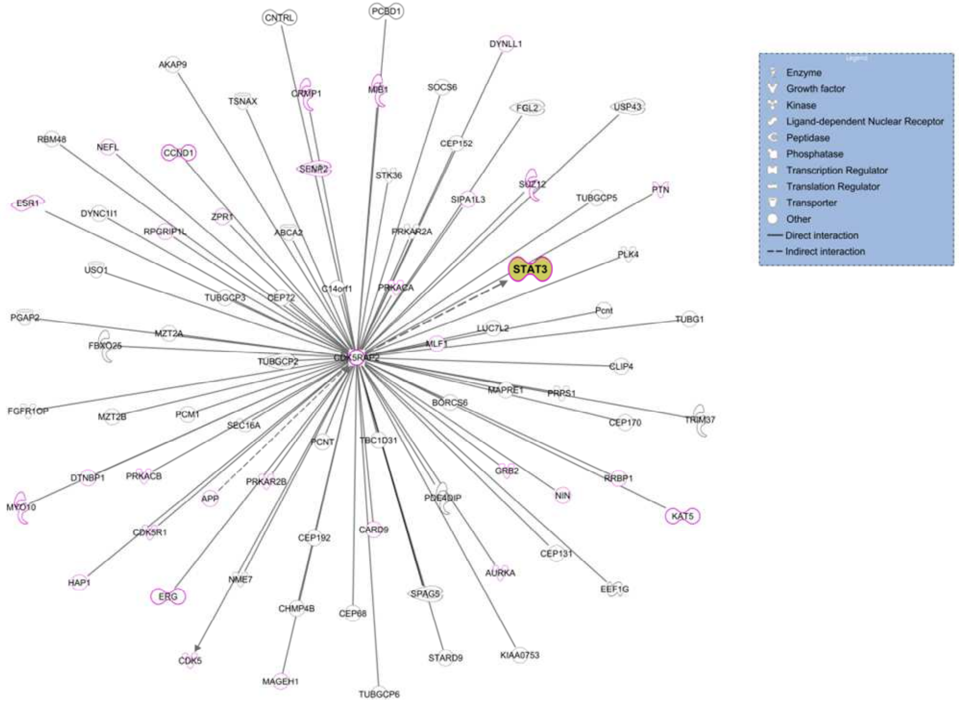

CHL1

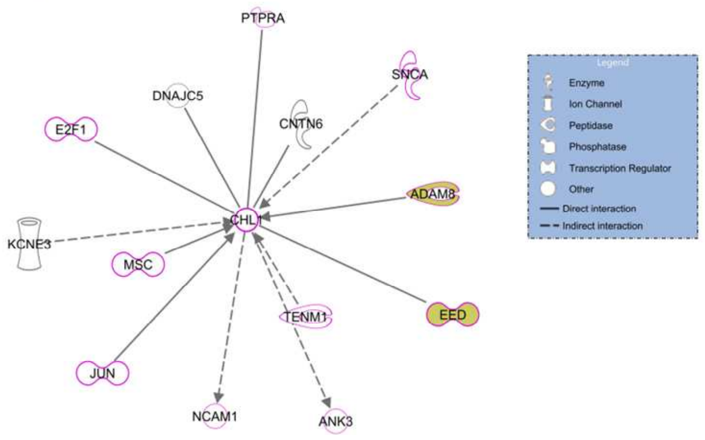

PPP2R2B

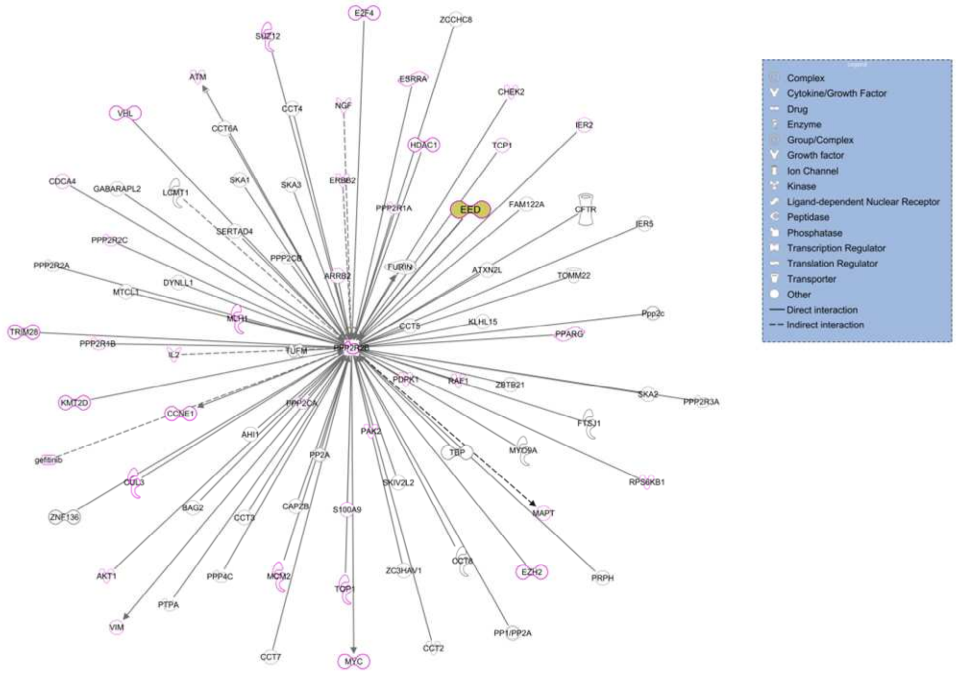

LITAF

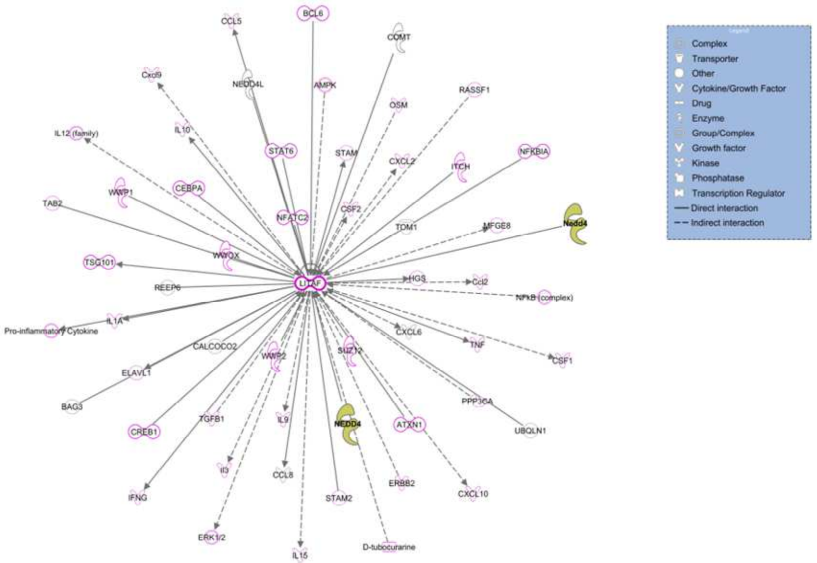

BBX

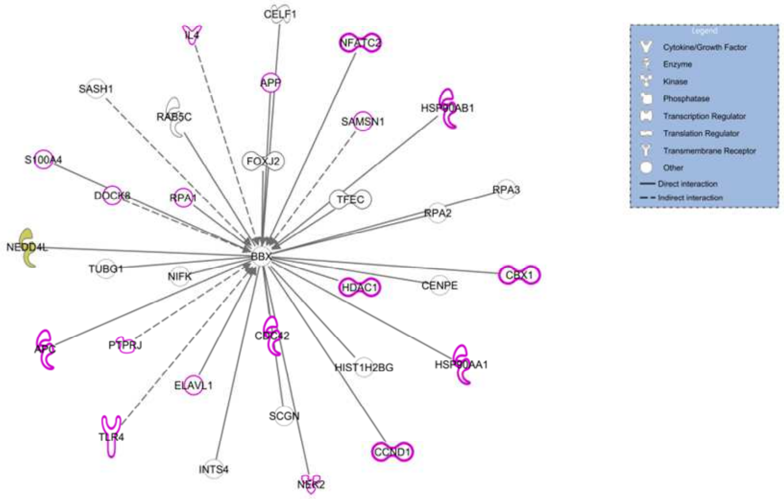

Figure S1: Gene interaction of DNMT3b target genes. Images obtained by the IPA tool. Relationships shown by straight lines represent; chemical-chemical interactions, chemical-protein interactions, correlation, protein-protein interactions and RNA-RNA interactions: non targeting interactions. Relationships shown by arrows represent; activation, causation, expression, localization, membership, modification, molecular cleavage, phosphorylation, protein-DNA interactions, protein-RNA interactions, regulation of binding and transcription, and the direction of the effect caused. The genes highlighted by IPA with category "cellular development" are labeled in pink and the genes selected by us are labeled in yellow.
